# Evaluation of the efficacy of cystinosin supplementation through *CTNS* mRNA delivery in experimental models for cystinosis

**DOI:** 10.1038/s41598-023-47085-w

**Published:** 2023-11-28

**Authors:** Tjessa Bondue, Sante Princiero Berlingerio, Florian Siegerist, Elena Sendino-Garví, Maximilian Schindler, Hans Jacobus Baelde, Sara Cairoli, Bianca Maria Goffredo, Fanny Oliveira Arcolino, Jürgen Dieker, Manoe Jacoba Janssen, Nicole Endlich, Roland Brock, Rik Gijsbers, Lambertus van den Heuvel, Elena Levtchenko

**Affiliations:** 1 Laboratory of Pediatric Nephrology, Department of Development and Regeneration, KU Leuven, Leuven, Belgium; 2 Institute of Anatomy and Cell Biology, Universitätsmedizin Greifswald, Greifswald, Germany; 3 Division Pharmacology, Utrecht Institute for Pharmaceutical Sciences, Utrecht University, Utrecht, The Netherlands; 4 Department of Pathology, Leiden University Medical Center, Leiden, The Netherlands; 5 Laboratory of Metabolic Biochemistry, Department of Pediatric Medicine, Bambino Gesù Children’s Hospital, IRCCS, Rome, Italy; 6 Department of Pediatric Nephrology, Emma Children’s Hospital and Emma Center for Personalized Medicine, Amsterdam UMC, Amsterdam, The Netherlands; 7 Mercurna B.V., Oss, The Netherlands; 8 Department of Medical Biosciences, Radboud University Medical Center, Nijmegen, The Netherlands; 9 Department of Medical Biochemistry, College of Medicine and Medical Sciences, Arabian Gulf University, Manama, Kingdom of Bahrain; 10 Laboratory for Molecular Virology and Gene Therapy, Department of Pharmaceutical and Pharmacological Sciences, KU Leuven, Leuven, Belgium; 11 Leuven Viral Vector Core (LVVC), KU Leuven, Leuven, Belgium; 12 Department of Pediatric Nephrology, Radboud University Medical Center, Nijmegen, The Netherlands; 13 Department of Pediatric Nephrology, Emma Children’s Hospital, Amsterdam UMC, H7-234, Meibergdreef 9, 1105AZ Amsterdam, The Netherlands

**Keywords:** Molecular medicine, Nephrology

## Abstract

Messenger RNA (mRNA) therapies are emerging in different disease areas, but have not yet reached the kidney field. Our aim was to study the feasibility to treat the genetic defect in cystinosis using synthetic mRNA in cell models and *ctns*^−/−^ zebrafish embryos. Cystinosis is a prototype lysosomal storage disorder caused by mutations in the *CTNS* gene, encoding the lysosomal cystine-H^+^ symporter cystinosin, and leading to cystine accumulation in all cells of the body. The kidneys are the first and the most severely affected organs, presenting glomerular and proximal tubular dysfunction, progressing to end-stage kidney failure. The current therapeutic standard cysteamine, reduces cystine levels, but has many side effects and does not restore kidney function. Here, we show that synthetic mRNA can restore lysosomal cystinosin expression following lipofection into *CTNS*^−/−^ kidney cells and injection into *ctns*^−/−^ zebrafish. A single *CTNS* mRNA administration decreases cellular cystine accumulation for up to 14 days in vitro. In the *ctns*^−/−^ zebrafish, *CTNS* mRNA therapy improves proximal tubular reabsorption, reduces proteinuria, and restores brush border expression of the multi-ligand receptor megalin. Therefore, this proof-of-principle study takes the first steps in establishing an mRNA-based therapy to restore cystinosin expression, resulting in cystine reduction in vitro and in the *ctns*^−/−^ larvae*,* and restoration of the zebrafish pronephros function.

## Introduction

Lysosomal storage disorders (LSD) are a group of over 70 monogenic diseases, affecting different lysosomal proteins and leading to lysosomal substrate accumulation^1^. Cystinosis is an autosomal recessive LSD caused by mutations in the *CTNS* gene (chr17.13), encoding for the cystine-H^+^ symporter cystinosin. Defective cystinosin results in intra-lysosomal cystine accumulation and recent research has demonstrated that cystinosin also regulates various metabolic and signalling pathways, including cell survival, nutrient sensing, and vesicle trafficking^2^. The kidneys are the first and the most severely affected organs, presenting defective proximal tubular reabsorption (Renal Fanconi Syndrome, RFS) and glomerular damage leading to end-stage kidney disease (ESKD)^3, 4^. Moreover, cystinosis also affects extra-renal organs, usually at adolescent or adult age^5^.

Since 1994, the standard therapy for cystinosis is the cystine-reducing agent cysteamine. While effective in prolonging kidney survival and postponing extra-renal complications, cysteamine does not reverse RFS and cannot correct the non-cystine transporting functions of cystinosin. Also, many side-effects and strict dosing schedules pose a significant burden on the patients^6^.

Preliminary results of gene therapy by transplanting *CTNS* cDNA containing lentiviral vector-transduced autologous hematopoietic stem cells (HSC) showed a reduction of tissue cystine accumulation and preserved eye, thyroid, and kidney function^7^. Nevertheless, HSC transplantation has several disadvantages, such as the risk of insertional mutagenesis and the need for leukapheresis and myeloablation^7, 8^.

Messenger RNA (mRNA-) based approaches have gained considerable attention during the SARS-CoV2 pandemic following the extensive use of mRNA vaccines^9–11^. Additionally, mRNA therapies are currently in pre-clinical and clinical trial phase for several genetic diseases, such as propionic acidaemia^12, 13^. mRNA acts in the cytosol, resulting in a fast translation of the therapeutic protein in dividing and non-dividing cells. Furthermore, due to the transient nature of mRNA expression, the treatment can be easily modulated or halted should adverse events occur^14^. mRNA can be modified to reduce immunogenicity and delivery vehicles can protect it from degradation in the bloodstream or allow for organ-specific targeting, if desired. De-immunization can be achieved either through elimination of sequence motifs that activate receptors of innate immunity or through incorporation of chemically modified bases that recapitulate modifications of endogenous mRNA. So far, mRNA-based therapies have been mainly restricted to targeting the liver^15, 16^.

In this study, we take the first steps in exploring the potential of mRNA-based therapy to treat cystinosis. We specifically focus on the kidney phenotype by studying the effect of the mRNA-treatment in 2D cultures of *CTNS*^−/−^ human kidney epithelial cells, proximal tubular epithelial cells cultured on a hollow fiber and in a *ctns*^−/−^ zebrafish cystinosis model. We opted for cystinosis as a prototypical disease to evaluate the potential of this approach, as cystinosis presents with the distinct phenotype of cystine accumulation offering a clear readout to evaluate the effectiveness of the treatment, and affects both podocytes and proximal tubular cells. At the organism level, the *ctns*^*−/−*^ zebrafish embryo was chosen as a stepping stone towards higher animal models as it enables to test the effectivity and potential toxicity of mRNA-based protein replacement after one-cell stage injection. Importantly, we demonstrate that a single delivery of sequence-optimized mRNA results in a reduction of cystine levels for up to 2 weeks in vitro, a finding with general relevance for the future treatment of other lysosomal storage disorders.

## Results

### *CTNS-3HA* mRNA transfection results in lysosomal cystinosin expression, reduces cystine levels and decreases in vitro epithelial leakage within 24 h post-transfection

**Figure 1:**
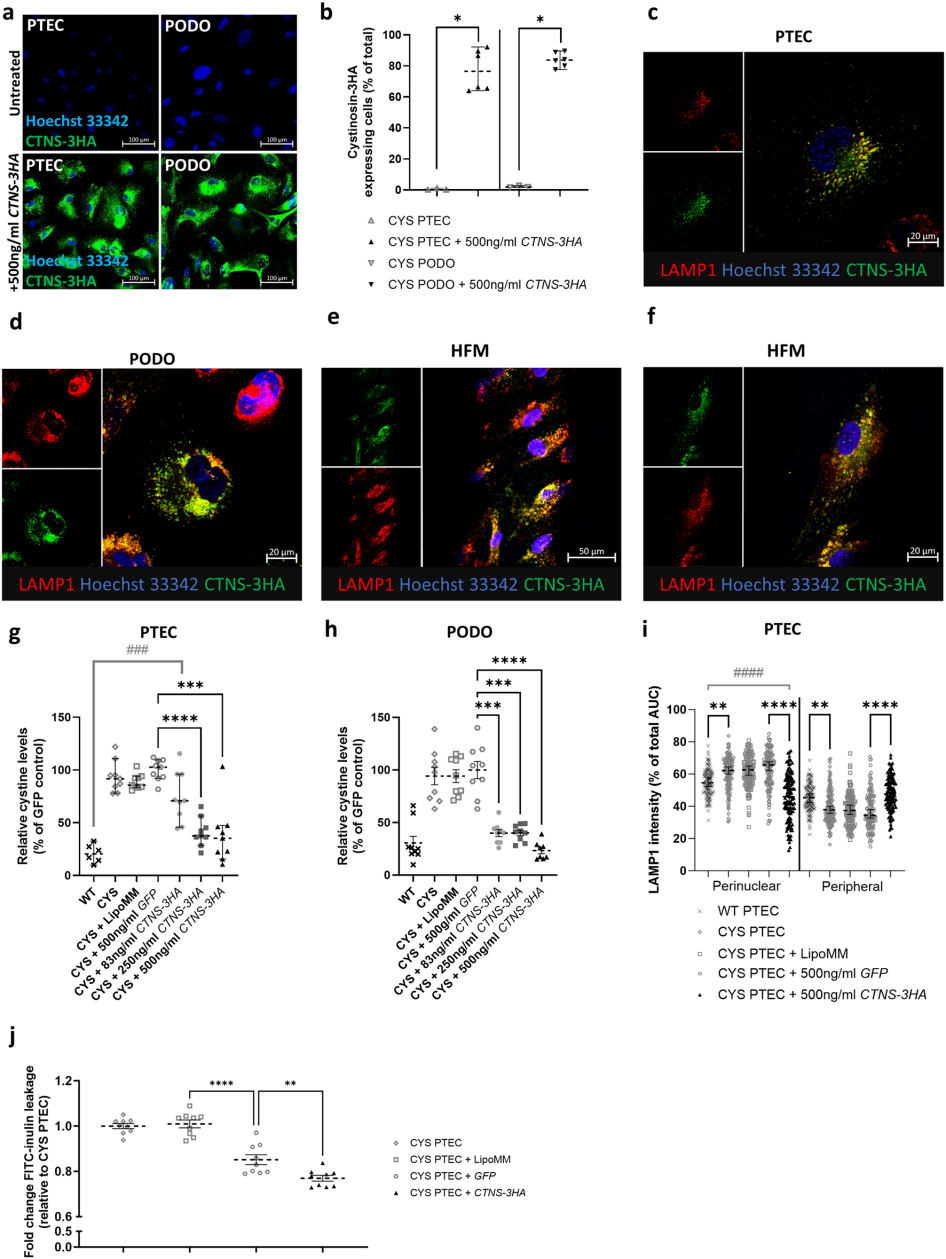
Transfection of cystinosis patient (CYS) derived proximal tubular epithelial cells (PTECs) and podocytes (PODOs) with *CTNS-3HA* mRNA results in lysosomal expression of cystinosin-3HA, reduction of cystine levels and reduced inulin leakage at 24 h post-transfection. (**a**) Cystinosin-3HA expression (green) after transfection of CYS PTECs and PODOs with 500 ng/ml *CTNS-3HA* mRNA (bottom) as compared with untreated cells (top). Scale bar = 100 µm. (**b**) Quantification of PTECs and PODOs expressing cystinosin-3HA in randomly acquired microscopical fields (each dot is one field from 3 independent experiments). Images were obtained using a Operetta CLS High Content Screening Microscope. Median and 95% CI are indicated. Data were analysed with Mann–Whitney tests. *p < 0.05. (**c–f**) Lysosomal localization was demonstrated at 24 h post-transfection by co-staining of cystinosin-3HA (green) with LAMP1 (red) in confocal images in a 2D cell model (**c,d**) (scale bar = 20 µm) and in PTECs on the hollow fiber membranes (HFM—**e,f**) (scale bar = 50 µm and 20 µm). (**g,h**) Reduction of lysosomal cystine accumulation in PTECs (**g**) and PODOs (**h**) at 24h post-transfection with increasing concentrations of *CTNS-3HA* mRNA. Cellular cystine levels were analysed by LC–MS. *CTNS-3HA* mRNA treated cells were compared with the *GFP* control using Kruskal Wallis test (PTECs) and Welch’s ANOVA (PODOs) (*). A second comparison between the transfected cells and wildtype control (Kruskal–Wallis—^#^) was performed. Median and 95% CI are represented for PTECs and mean with SEM for PODOs (n = 3 independent experiments). *p < 0.05; ***^,###^*p* < 0.001; ****p < 0.0001. (i) At 24 h post-transfection, cells treated with *CTNS-3HA* mRNA show a reduced perinuclear LAMP1 signal (% of total, Kruskal–Wallis test—*). The (peripheral) distribution in the *CTNS-3HA* treated PTECs was comparable to the wildtype control (Kruskal–Wallis test—^#^) (n = 120 cells). Median with 95% CI is indicated. **p < 0.01; ****^,####^*p* < 0.0001. (**i**) The fold change leakage of FITC-inulin (relative to untreated PTECs) was evaluated in PTECs cultured on the hollow fiber membranes at 24 h post-transfection (n = 3 biological replicates). Data were evaluated with an one-way ANOVA. Mean and SEM are shown. **p < 0.01; ****p < 0.01.

Proximal tubular epithelial cells (PTECs) and podocytes (PODOs) from cystinosis patients (CYS) were transfected with 500 ng/ml of *CTNS-3HA* mRNA to study transfection efficiency and protein localization at 24 h (24 h) post-transfection. The protein coding sequence of the mRNA was optimized for protein expression through removal of potential immune-stimulating motifs and incorporation of untranslated regions (UTRs) that were selected for high protein expression. Protein expression was detected in 76.3% [64.0%, 92.2%] [95% CI] of CYS PTECs and 83.8% [77.6%, 89.6%] of CYS PODOs (Fig. 1a,b). Cystinosin is a protein expressed in the endo-lysosomal membranes and acts as a cystine-H^+^ symporter. Co-staining for the lysosomal associated membrane protein 1 (LAMP1) confirmed the presence of the CTNS-3HA protein in the lysosomal compartment (Fig. 1c,d) at 24 h post-transfection. Also, in the 3D bioengineered kidney tubules, generated by the culturing of the CYS PTECs on the hollow fiber membranes, lysosomal protein expression could be shown at 24 h post-transfection (Fig. 1e,f).

To determine whether mRNA-mediated cystinosin expression had a functional effect, cystine levels were quantified in CYS PTECs and CYS PODOs at 24 h post-transfection with increasing concentrations of *CTNS-3HA* mRNA. The CYS PTECs transfected with 250 ng/ml or 500 ng/ml of *CTNS-3HA* mRNA showed a decrease in cellular cystine to 37.5% [28.3%, 56.4%] and 35.3% [15.5%, 47.4%] of the levels observed in the CYS PTECs transfected with *GFP* mRNA, respectively. These levels were comparable to those observed in the wildtype reference PTECs and underline the fast-acting nature of the mRNA (Fig. 1g). The lower concentration of *CTNS-3HA* mRNA (83 ng/ml *CTNS-3HA*) showed a less pronounced decrease in cystine levels (70.8% [45.8%, 96.1%] of the *GFP* control) and a smaller proportion of cells with detectable protein (Supplementary Table S1). Similar results were obtained in CYS PODOs (Fig. 1h), but with all tested mRNA concentrations leading to cystine concentrations approaching wildtype levels.

Additionally, the LAMP1 staining was used to evaluate the effect of the mRNA on the previously described perinuclear accumulation of lysosomes in CYS PTECs^4^. In the cystinotic cells, the perinuclear signal of LAMP1 was significantly (*p* < 0.01) increased to 62.19% [60.0%, 64.5%] of the total signal, as compared with the wildtype PTECs that only show 54.65% [52.3%,57.7%] of the total LAMP1 in this area. We could demonstrate an amelioration of the lysosomal distribution, characterized by less perinuclear (45.93% [42.0%, 50.7%]) accumulation of LAMP1 in favour of increased peripheral localization in cells treated with *CTNS-3HA* mRNA (Fig. 1i). Finally, we also evaluated the leakage of FITC-inulin after perfusion of the hollow fiber tubular structures that were mounted in a 3D-printed chamber, as previously described^17^. Here, we observed that the transfection itself (+ *GFP* mRNA) had a substantial effect on the leakage of inulin, with a leakage fold change (relative to the untreated reference) of 0.85 (± 0.02) (SEM). However, the hollow fibers that were transfected with *CTNS-3HA* mRNA presented an additional significant (*p* < 0.01) improvement, with leakage fold change further decreasing to 0.76 (± 0.01) (Fig. 1j).

### *CTNS-3HA* mRNA transfection results in detectable cystinosin-3HA protein expression for up to 4 days and cystine reduction for at least 10 days

**Figure 2:**
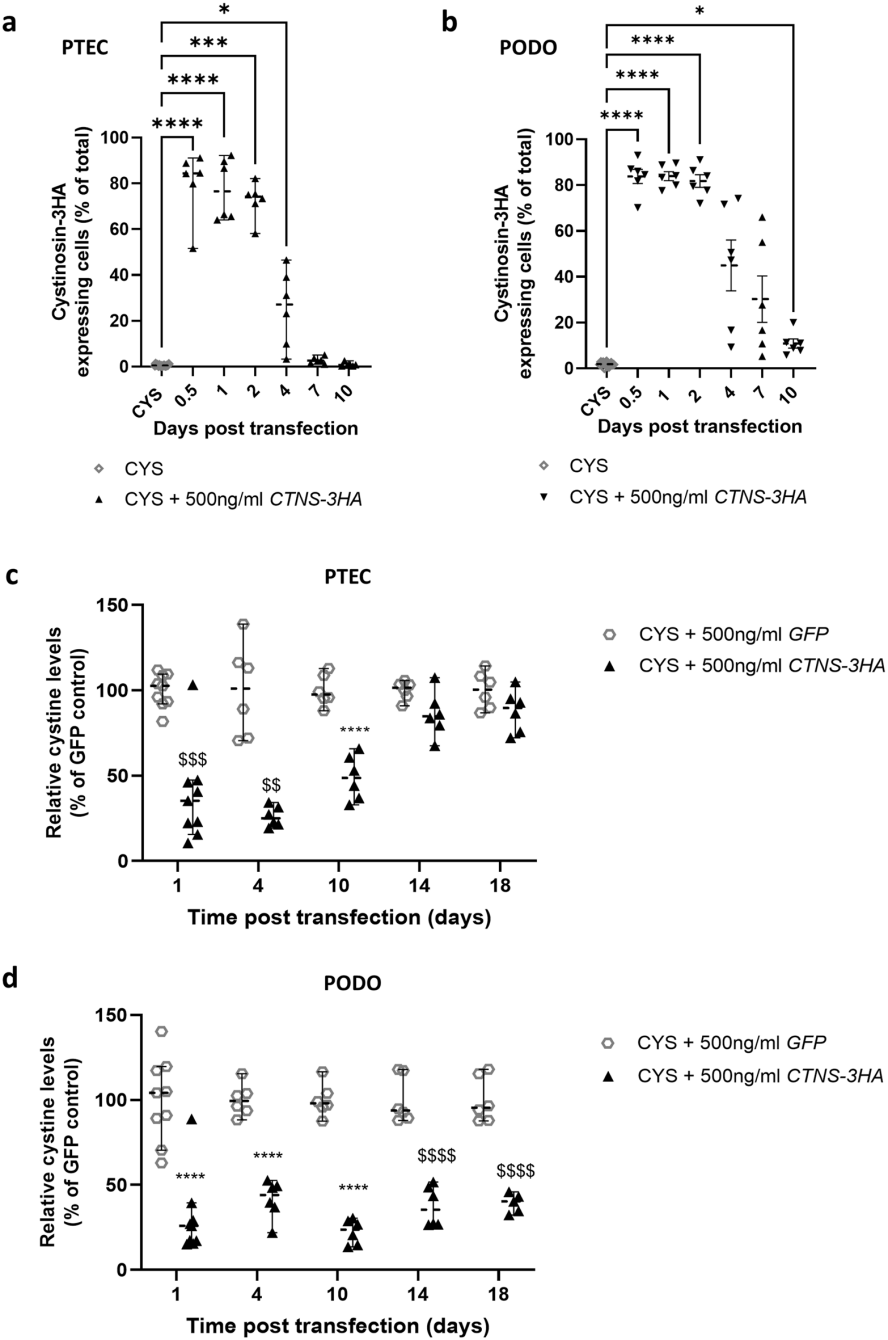
Transfection of cystinosis patient (CYS) derived proximal tubular epithelial cells (PTECs) and podocytes (PODOs) results in functional cystinosin-3HA expression for up to 4 and 10 days, and reduces cystine levels for up to 10 and 18 days, respectively. (**a,b**) Time course of cystinosin-3HA expression in PTECs (**a**) and PODOs (**b**). Images were obtained using the Operetta CLS High Content Screening Microscope and percentage of cells with detectable cystinosin-3HA expression quantified (each dot represents one observed field from 3 independent experiments). Data were analysed using a Kruskal Wallis test (PTECs) and Welch’s ANOVA test (PODOs). Median and 95% CI are represented for PTECs, and mean and SEM are shown for the PODOs. *p < 0.05; ***p < 0.001; ****p < 0.0001. (**c,d**) Time course of cystine reduction in CYS PTECs (**c**) and PODOs (**d**) that were transfected with *CTNS-3HA* mRNA. Cystine levels were measured at 4, 10, 14 and 18 days post-transfection and were expressed as percentage (%) of levels measured in cells transfected with *GFP* mRNA. Each dot represents a replicate, coming from 2 independent transfections. Statistical significance was evaluated using individual tests for each time point, one-way ANOVA (****p < 0.001) and Kruskal Wallis test (^$$^*p* < 0.01; ^$$$^*p* < 0.001; ^$$$$^*p* < 0.0001). Median and 95% CI are indicated for all time points.

Next, we assessed the duration of cystinosin expression following transfection. Cystinosin-3HA expressing CYS PTECs were evaluated from 12 h to 10 days post-transfection, with the number of cells with detectable cystinosin ranging from 84.4% [51.6%, 91.1%] at 12 h to 27.1% [3.3%, 46.5%] after 4 days. At 7 and 10 days, only a few cystinosin expressing CYS PTECs could be detected (2.6% [1.0%, 5.0%] and 0.7% [0.3%, 2.5%], respectively) (Fig. 2a). In CYS PODOs, the fraction of positive cells ranged from 83.8% (± 3.1) at 12 h to 45.0% (± 11.1) at 4 days, and a considerable number of cells positive for cystinosin remained detectable at 7 (30.25% (± 10.1)) and 10 days (10.85% (± 2.0)) (Fig. 2b).

Given the reduction in cystine levels at 24 h post-transfection and detectable cystinosin protein for up to 4 days, we evaluated the long-term effect on cystine levels. Cystine levels were reduced in CYS PTECs as compared with the *GFP* mRNA control for 10 days, reaching pre-treatment levels only after 14 days (Fig. 2c). *CTNS* mRNA transfected CYS PODOs maintained low cystine levels for up to 18 days, with values ranging between 23.3% (± 3.0) and 40.3% [32.4%, 45.9%] of *GFP* treated cells (Fig. 2d).

### ***CTNS-mCherry*** mRNA injection results in cystinosin-mCherry protein expression in ***ctns***^−/−^ zebrafish larvae

**Figure 3:**
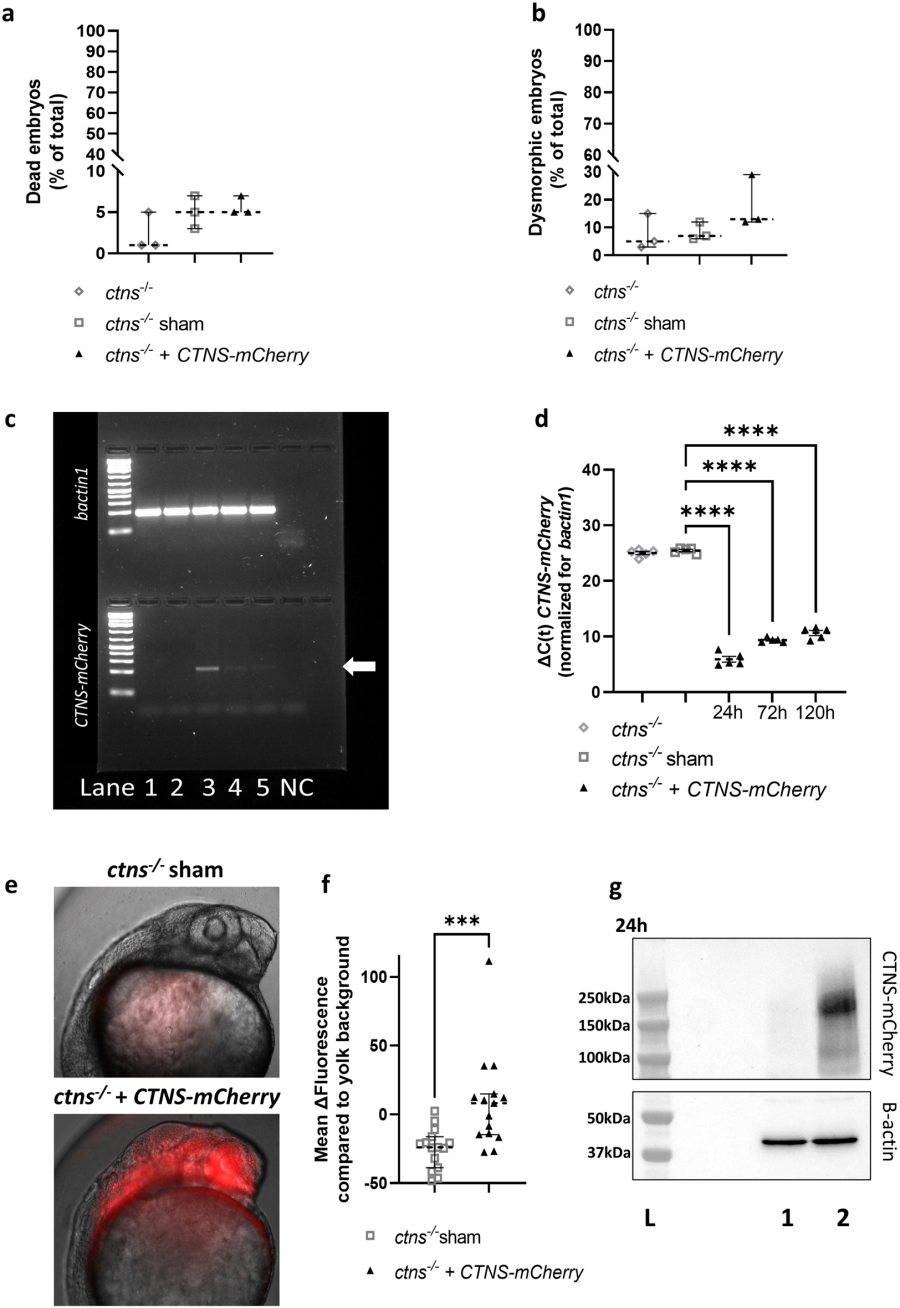
Injection of human *CTNS-mCherry* mRNA in fertilized eggs of *ctns*^−/−^ zebrafish results in embryonic protein expression and does not cause toxicity. (**a,b**) The percentage (%) of dead (**a**) and dysmorphic embryos (**b**) was quantified at 5 days post-injection of human *CTNS-mCherry* (30–150 embryos per experiment, n = 3). Statistical analysis was done by means of a Kruskal Wallis test. Median and 95% CI are indicated. (**c,d**) *CTNS-mCherry* mRNA levels were quantified in the untreated (lane 1) and sham injected (lane 2) fish at 24 h (lane 3), 72 h (lane 4) and 120 h (lane 5) post-injection by quantitative RT-PCR (**d**) and agarose gel electrophoresis (arrow—**c**). Expression of *bactin1* mRNA was used for normalization. Per condition, ΔC(t) is shown for each biological replicate (n = 5) and analysis was done with an one way ANOVA. Untreated and sham group C(t)s were artificially put at 40 cycles for quantification and mean and SEM are shown. ****p < 0.0001. (**e,f**) Protein expression was quantified at 24 h post-injection by live in vivo microscopy. Mean mCherry-fluorescence per embryo was quantified in a total of 15 embryos and the yolk background subtracted. Images were obtained from the larvae head region using the Olympus IX71 widefield fluorescence microscope. Statistical analysis was performed with a Mann–Whitney test and median and 95% CI are indicated. ***p < 0.001. (**g**) Expression of cystinosin-mCherry protein at 24 h was confirmed in *CTNS-mCherry* injected fish (lane 2) in comparison with the sham injected control (lane 1) by a western blot (top) with beta-actin as the loading control (bottom). Original blots and gel are presented in Supplementary Figs. S4 and S5. *L*=ladder.

After demonstrating the effectiveness of synthetic *CTNS* mRNA in vitro, we aimed to validate the mRNA-based strategy in the *ctns*^*−/−*^ zebrafish^18^ by directly injecting human *CTNS-mCherry* mRNA into one-cell embryos. Neither the injection procedure (sham), nor the treatment with *CTNS-mCherry* mRNA resulted in significant embryonic lethality or embryo dysmorphism, assessed as the proportion of larvae with pericardial oedema or an abnormally curved spine (Supplementary Fig. S1a,b) at 120 h post-injection (Fig. 3a,b).

*CTNS-mCherry* treated embryos showed detectable levels of *CTNS* mRNA (Fig. 3c,d) for up to 5 days and protein expression was confirmed at 24 h post-injection by fluorescence microscopy in the live embryo (Fig. 3e,f) and with western blotting (Fig. 3g). Notably, the mCherry signal was detectable in the live embryo within 6h post-injection and persisted for up to 48 h (Supplementary Fig. S2a,b). Additionally, cystinosin-mCherry expression could be shown by anti-mCherry immunostaining at 72 h post-injection as well (Supplementary Fig. S2c,d).

### ***CTNS-mCherry*** mRNA injection reduces cystine accumulation in ***ctns***^−/−^ zebrafish larvae and improves the pronephros function

The* ctns*^*−/−*^ zebrafish larvae are known to accumulate cystine (Supplementary Fig. S3a) and suffer from reduced tubular reabsorption (Supplementary Fig. S3b), measured by a reduced uptake of a labelled low-molecular weight dextran molecule by the proximal tubule. The tubular multi-ligand receptor megalin is responsible for the uptake of a wide array of substances. A reduced surface expression of the receptor was observed in the tubular segment of *ctns*^*−/−*^ larvae at 72 h, which can be directly related to the observed tubular reabsorption deficiency (Supplementary Fig. S3c). Furthermore, *ctns*^*−/−*^ zebrafish suffer from glomerular dysfunction. Proteinuria can be quantified in *[Tg(l-fabp:DBP:eGFP)]* fish that express a GFP-tagged version of the high-molecular weight vitamin D binding protein (DBP-GFP)^19^. The GFP-labelled DBP is not able to pass a healthy glomerular filtration barrier and results in high levels of green fluorescence in healthy embryos. In this study, we used a *ctns*^*−/−*^*[Tg(l-fabp:DBP:eGFP)]* fish model for cystinosis that shows a 15% reduction (mean) in green fluorescence at 5 days post-fertilization as compared with the *ctns*^+*/*+^*[Tg(l-fabp:DBP:eGFP)]* larvae, indicative of an increase in DBP-GFP protein loss in the fish water (Supplementary Fig. S3d). This decrease in DBP-GFP fluorescence was shown to be independent from the liver DBP-GFP production (Supplementary Fig. S3e).

**Figure 4:**
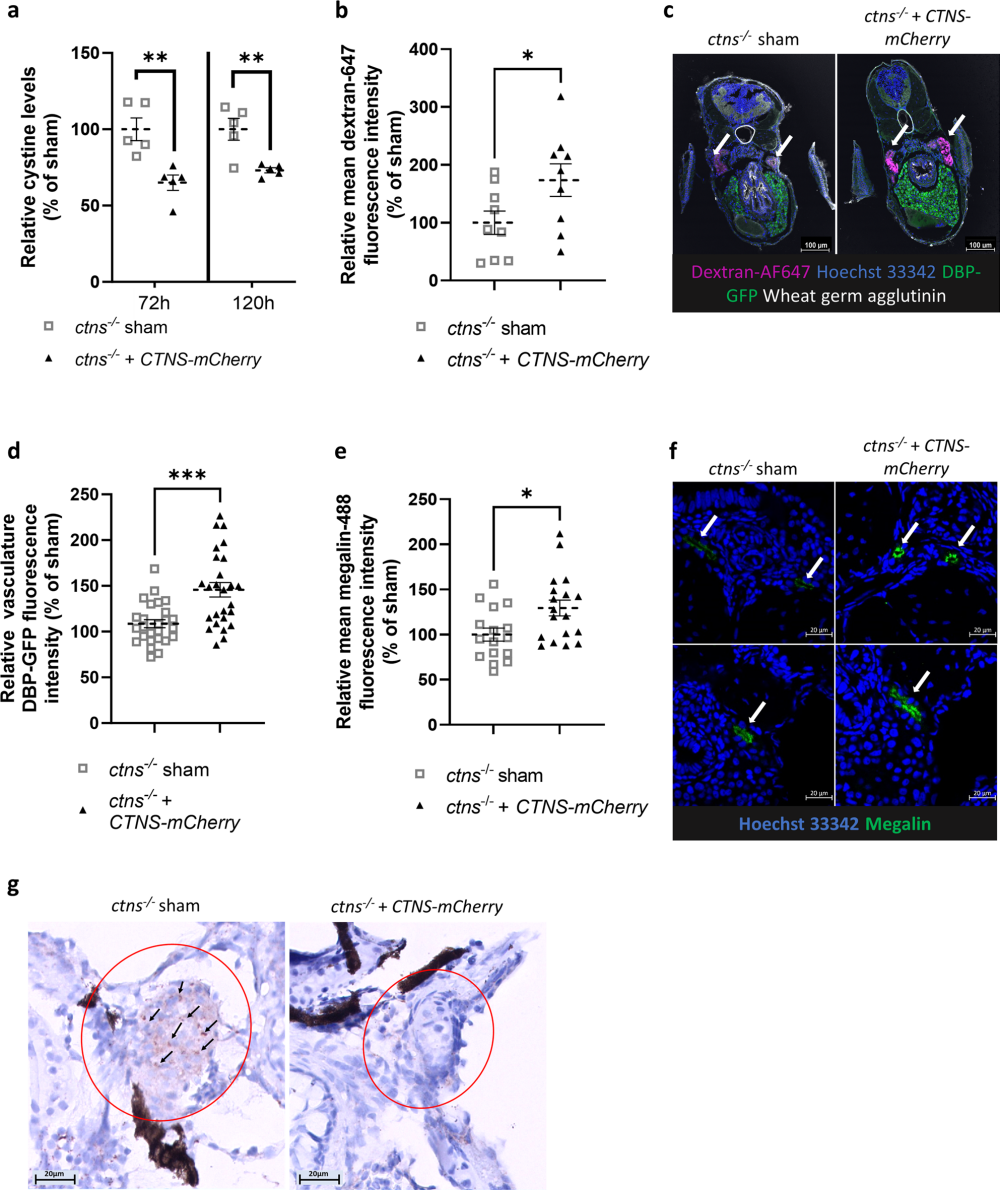
Injection of human *CTNS-mCherry* mRNA in fertilized eggs of *ctns*^−/−^ zebrafish decreases cystine accumulation, restores tubular reabsorption and alleviates glomerular proteinuria. (**a**) Injected *ctns*^−/−^ zebrafish larvae were collected in groups of 10–12 fish. Cystine levels in treated larvae were measured at 72 h and 120 h post-injection (n = 5 groups). Statistical analysis was performed using a Student’s t-test. Mean and SEM are indicated. **p < 0.01. (**b,c**) mRNA treated *ctns*^*−/−*^*[Tg(l-fabp:DBP:eGFP)]* larvae were injected with 10 kDa dextran-AF647 for evaluation of low molecular weight proteinuria (LMWP) at 72 h post-injection and fixed for cryosection after 16 h. Sections were stained with Hoechst 33342 (blue) and wheat germ agglutinin (WGA, white), with the DBP-GFP visible in the liver (green). Tubular intensity of dextran-AF647 fluorescence (magenta) was measured and used to quantify low molecular weight protein absorption. Data were analysed with a Student’s t-test and mean and SEM are indicated (n = 9 fish). Scale bar = 100 µm. *p < 0.05. (**d**) Glomerular proteinuria was evaluated in the *ctns*^*−/−*^*[Tg(l-fabp:DBP:eGFP)]* larvae at 120 h post-injection of *CTNS-mCherry* mRNA. Images were generated with the Acquifer imaging machine. For quantification, an automatized FIJI script was used within a mask determined by segmentation of blood vessels upon traversing blood cells. Data were analysed with a Welch’s t-test. Mean and SEM are indicated (n = 26 fish). ***p < 0.001. (**e,f**) Sections of injected *ctns*^−/−^ fish were prepared at 72 h post-injection and stained for the megalin multi-ligand receptor on the proximal tubular brush border (green), with a nuclear counterstain (blue). Confocal images were obtained and Alexa-488 intensity was measured in each tubule (n = 8 fish) and showed the restauration of proximal tubular megalin expression in treated larvae. Statistical significance was tested using an Student’s t-test and mean and SEM are indicated. *p < 0.05. Scale bar = 20 µm. (**g**) Paraffin sections of embryos at 120 h post-injection were stained for cleaved caspase-3 and the proximal tubulus identified (red circle). High signal spots are accentuated by an arrow. Scale bar = 20 µm.

To show the functional effect of the *CTNS-mCherry* mRNA injection, cystine levels were measured in *ctns*^*−/−*^ zebrafish larvae at 72 h and 120 h after one-cell stage injection. Cystine accumulation decreased to 65.0% (± 5.1) at 72 h and 73.1% (± 1.7) at 120 h as compared with the sham-treated fish (Fig. 4a), demonstrating the potential of the therapeutic approach.

With this substantial effect on cystine accumulation, we then assessed the effect of the mRNA treatment on the kidney phenotype. First, low molecular weight dextran was injected at 72 h to assess proximal tubular reabsorption and showed a significant (*p* < 0.05) improvement in the tubular uptake (Fig. 4b,c). This was also associated with increased tubular expression of megalin at 72 h post-injection (Fig. 4e,f). Subsequently, we evaluated the potential effect of the mRNA-based treatment on proteinuria in the *ctns*^*−/−*^*[Tg(l-fabp:DBP:eGFP)]* larvae^18, 19^. Here, one-cell stage mRNA injection reduced proteinuria at 120 h post-injection (Fig. 4d), as shown by an increase in embryo DBP-GFP fluorescence by 45.7% (mean) without significant changes in the liver DBP-GFP fluorescence (Supplementary Fig. S3f). Finally, we could also show a reduced number of apoptotic spots in the tubular segment after staining for cleaved caspase-3 in *CTNS-mCherry* treated embryos at 120 h post-injection (Fig. 4g).

Taken together, these results demonstrate that *CTNS* mRNA-based cystinosin expression is feasible and effective in reducing intracellular cystine. Furthermore, the *CTNS* mRNA supplementation in the early stage zebrafish embryo improved the tubular reabsorption and reduced proteinuria. These results illustrate that *CTNS* mRNA-based therapy may represent a promising approach for the treatment of cystinosis.

## Discussion

Since the discovery of the *CTNS* gene, efforts to develop a gene replacement therapy for cystinosis have been undertaken^20^. Transplantation of *CTNS-*transduced autologous HSCs is efficient in reducing cystine accumulation and correcting different cellular pathways^8^. However, this therapy requires leukapheresis, myeloablation and harbours other risks that are not expected for mRNA-based approaches^7, 8, 12^. We studied the potential of *CTNS* mRNA therapy to ameliorate cystinosis, a monogenic lysosomal storage disorder having a well-defined phenotype, presenting with cystine accumulation and both podocyte and proximal tubular dysfunction in the kidney. We applied a synthetic in vitro transcribed mRNA, with modified codon usage to reduce immunogenicity^15^, that was shown to not be toxic in the tested in vivo zebrafish embryo model. Moreover, in vitro transcribed mRNA is suitable for human applications and is currently being tested in clinical trials for propionic acidemia (NCT04159103 and NCT05130437), cystic fibrosis (NCT05668741), methylmalonic acidemia (NCT04899310 and NCT05295433), ornithine transcarbamylase deficiency (NCT05526066) and glycogen storage disease (NCT05095727)).

A major advantage of the mRNA-based approach is the rapid expression of the protein after introduction of the mRNA into the host system^15^. Indeed, we were able to confirm lysosomal protein expression within 12 h in human cultured cells and within 6 h post-injection in the zebrafish embryos. As expected, mRNA delivery resulted in a transient expression of the protein, which offers dosing flexibility and enhances safety because of the reversibility of any potential side effects. Moreover, mRNA therapy has no risk of insertional mutagenesis^15^. On the other hand, the transient nature of expression can also be considered a disadvantage, as this therapy will require repeated dosing. Another potential limitation of mRNA can be the initiation of an immune response due to the recognition of the mRNA by the innate immune receptors (such as TLR7 and RIG-I)^15^. The immunogenicity of our mRNA was reduced through codon optimization and dsRNA removal. While the immunogenicity of mRNA was less relevant for the cell studies, the expression of TLR7 and RIG-I orthologues has been demonstrated in zebrafish^21^. While our experiments and previous studies using the injection of human mRNA in fish did not show any toxicity^22^, only innate immune responses can be taken into account in the zebrafish model. Whether adaptive reactions can also occur, will be heavily influenced by the delivery vehicle, which will be the subject of future research and will require mammalian models^23^. The stability of mRNA can be further improved through careful selection of UTRs and as poly-A tail shortening destabilizes the RNA with every translation round, a sufficiently long (150A) poly-A tail was chosen^24, 25^. After mRNA-degradation, protein stability is the main determinant for the duration of the therapeutic effect^12, 15^. Lysosomal transporters are degraded in intraluminal vesicles during lysosomal fusion^26^. Notably, Nevo et al. showed that the turnover rate of cystinosin-GFP in fibroblasts mainly depends on the dilution of the protein during cell division, with cystinosin itself being very stable^27^. In our study we could detect cystinosin-3HA protein for up to 4 days in the kidney epithelial cells. The *CTNS-3HA* mRNA used in this study was equipped with standard translation-promoting UTRs, but the identification of UTRs tailored to the specific cell types could further prolong the duration of protein expression. Cystinosin-mCherry was detectable for up to 72 h post-injection using immunostaining in the live zebrafish embryo. The faster decrease in detectable protein is likely caused by mRNA dilution due to the rapid cell division (from 1 to 1000 cells within 3 h) during early embryo development, but will also depend on the sensitivity of the specific methods for detection, which differed between the in vitro and in vivo studies.

Cystine accumulation, as the main hallmark of cystinosis, is the most quantifiable readout for new therapies in this disorder. Strikingly, following a single *CTNS* mRNA transfection, we showed a decrease in cystine levels within 24 h, approaching wildtype levels, and lasting for up to 14 days (PTECs) and 18 days (PODOs). This timeframe is in line with that observed in preclinical research towards mRNA-based therapies for other monogenic diseases (for example, cystic fibrosis)^28^. Notably, we observed that the cystinotic PTECs required higher mRNA doses (> 83 ng/ml) and maintained reduced cystine levels for a shorter time period as compared with the cystinotic PODOs, potentially related to the higher endocytosis rate and metabolic activity of PTECs. Decreased cystine levels were observed in the zebrafish model for at least 120 h post-injection. Here, wildtype levels were not reached, potentially attributed to the rapid dilution of the protein in the embryo after one-cell stage injection. Importantly, the time course of substrate depletion exceeded that of detectable protein in both the in vitro and in vivo models, a phenomenon that was previously shown in the study of mRNA-based therapies for Fabry disease^29^. Therefore, we hypothesize that low levels of protein expression can still have a functional effect, which also has important implications for the future investigation of a feasible dosing regimen. For a full evaluation of a therapy, a suitable delivery vehicle should be identified first and the immunogenicity of the packaged mRNA evaluated in a rodent model. Both of these factors will greatly influence the future dosing schedule.

The current standard treatment of cystinosis, cysteamine, is not able to cure the RFS and can only delay the onset of ESKD. The inability of cysteamine to reverse RFS has been explained by the loss of proximal tubular cells due to enhanced apoptosis^30^. Moreover, it has been shown that cystinosin plays a direct role in other functions (regulating vesicle trafficking, autophagy and redox homeostasis) in proximal tubular cells and therefore, the mere reduction of intracellular cystine is insufficient to restore proximal tubular reabsorption^2, 31–33^. Notably, Festa et al*.* found that abnormal proximal tubule dedifferentiation resulted in reduced expression of apical transporters and caused RFS in cystinosis. This phenotype was only restored after adenoviral re-expression of *CTNS,* and not by cysteamine^32^*.* In our zebrafish larvae, we demonstrated that early-stage *CTNS-mCherry* mRNA delivery improved proximal tubular uptake of low molecular weight dextran, a test for proximal tubular reabsorption, and reduced overall proteinuria. In addition, we studied the expression of megalin, a multi-ligand receptor, responsible for the reabsorption of low molecular weight proteins, hormones, and other polypeptides. Here, we found that treatment with *CTNS* mRNA resulted in a mild increase in receptor expression, which might indicate an overall improvement of diverse cellular pathways impaired in cystinosis proximal tubules^34–36^. Finally, we could also demonstrate a mild reduction of cleaved caspase-3, indicative of a reduced rate of apoptosis and the potential of the mRNA-based therapy to reduce tubular epithelial cell loss^18^.

While recent studies for monogenic diseases have shown the potential of mRNA-based therapies^12, 28, 37^ genetic kidney diseases lag behind^15^. For treating the kidneys, a suitable kidney-targeting delivery vehicle that allows systemic injection of mRNA needs to be developed. Our study used a direct injection of naked mRNA into fertilized eggs of the zebrafish and provided proof-of-principle to establish effectivity of exogenously delivered mRNA. The zebrafish is well suited for the high-throughput testing of mRNA-based therapeutics, as the small size and optical transparency allows for a cost-effective way to assess protein expression in the live embryo by means of a mRNA encoding a fluorescent (fusion) protein. Additionally, the large number of embryos per fertilization round and simplicity of the one-cell stage injections allow for the high-throughput study of effectivity, which is disease specific^38, 39^. Therefore, the zebrafish can be seen as a stepping stone towards studies in rodent models, where further exploration of targeting capabilities and administration routes, which are species specific, take centre stage. Moreover, using zebrafish allows researchers to reduce the number of the rodents for testing mRNA-therapeutics to meet the general 3R (reduction, replacement, refinement) principles of animal research. However, the zebrafish model also has limitations. As we injected mRNA at the one-cell stage, this study was not suitable to evaluate the effect of the therapy on the fertility or hatching rate, nor to study the potential dosing regimens as the mRNA is quickly diluted in the dividing embryo. In addition, studying zebrafish larvae does not allow for prediction of the full immunogenic response, as only innate immunity responses can be studied in the zebrafish larvae^23^. In the future, a full safety study will have to be performed in a rodent model, after identification of the proper delivery vehicle, which will also greatly influence safety.

Notably, HSC-based gene therapy for cystinosis demonstrates that HSCs can differentiate into macrophages that can produce tunnelling nanotubes. These tunnelling nanotubes are subsequently used to transfer cystinosin-bearing lysosomes to other cells^40^. This illustrates that *CTNS* correction can work in trans and that it might be possible for *CTNS* expression in the bone marrow or in the liver to also benefit the kidney. However, the ultimate goal should be to develop a kidney targeting delivery vehicle, which protects the mRNA from degradation in the bloodstream and reduces immunogenicity by mRNA shielding^15^. At this moment, no kidney-targeting delivery vehicles for mRNA have been described, with the targeting of the proximal tubule being the biggest challenge, as the size of the encapsulated mRNA does not allow for passage through the glomerular filtration barrier (GFM). Nevertheless, filtration of mRNA formulations might be possible in patients with compromised podocyte function, as is the case in cystinosis and other kidney diseases. Furthermore, the basolateral uptake of 400 nm nanoparticles in PTECs has been shown^15, 16, 41^. In our study, we could already demonstrate a successful lipofectamine-mediated transfection in a hollow fiber cultured proximal tubule mode, mimicking basolateral delivery of mRNA. Finally, it must be noted that cystinosis is a systemic disease and an effect of the mRNA treatment on other organs requires further study. Currently, mRNA-delivery vehicles have been used for corneal treatment (topical administration)^42^, which might be of interest for cystinosis. Moreover, the liver, lungs and spleen can be targeted by mRNA delivery vehicles^43^. In the case of cystinosis, it will be important to evaluate if multiple organs can be reached by a single delivery vehicle or a mix of different delivery vehicles, equipped with multiple targeting molecules will be required.

In conclusion, our study is the first step to establishing mRNA-based therapy for cystinosis and paves the way for mRNA therapeutics in other genetic kidney diseases. Ongoing studies will focus on the degree of correction that can be achieved with expression in the liver, which is the main target organ for current mRNA delivery systems. In parallel, the search for kidney-targeted delivery vehicles that would allow for specific mRNA delivery to the kidney cells after systemic injection, is ongoing. Next to the delivery vehicle in a rodent model, special attention will be paid to the dosing frequency and the adaptive immune response.

## Materials and methods

### Cell culture

Conditionally immortalized proximal tubular epithelial cells (PTECs) and podocytes (PODOs), derived from healthy donors (WT) and cystinosis (CYS) patients (homozygous 57 kb deletion)^3, 44–46^, were cultured in DMEM F-12 (Biowest), as described before^4, 47^. The hollow fiber membrane (HFM) 3D model is representative of a functional kidney tubule and was prepared by seeding PTECs on L-DOPA and Collagen-IV coated fibers at 33 °C, as described^17^. Experiments were performed after differentiation at 37 °C for 10 (PTECs) or 12 days (PODOs).

The podocyte cell lines were generated by previous studies with protocols that were in accordance with the Declaration of Helsinki and approved by the Ethics Committee of the University Hospitals Leuven (S54695)^3^. The PTEC cell lines used in this study were developed according to the medical ethical guidelines and previously published by Wilmer et al.^44^. For the generation of all of these cells, informed consent was obtained from the donors.

### mRNA synthesis

Human *CTNS* mRNAs (based on NM_004937.3) with C-terminal 3xHA (amino acid sequence: YPYDVPDYASYPYDVPDYAYPYDVPDYA) or mCherry-tag (KM983420.1) were synthesized by RiboPro B.V. (Oss, The Netherlands) and de-immunized via codon optimization and dsRNA reduction. Each mRNA was equipped with translation promoting 5ʹ and 3ʹ untranslated regions (UTRs), a 5ʹ anti-reverse capping analogue (ARCA), and a 150A-poly-A-tail. mRNA quality was assessed by spectrophotometry and gel electrophoresis.

### mRNA transfection

0.15 µl of Lipofectamine MessengerMAX (LipoMM—Thermofisher) was mixed with 5 µl of Opti-MEM medium (Gibco) per sample and incubated for 10 min (min) at room temperature (RT). The solution was mixed by pipetting 5 µl of Opti-MEM per 50 ng of mRNA, incubated for 5 min at room temperature (RT) before transfection and added to the cells (1/10 total volume).

### Transfection efficiency and protein localization

Transfected cells were fixed with 4% paraformaldehyde (PFA-Alpha Aesar), permeabilized with 0.1% Triton X-100 (Sigma) and blocked in 2% bovine serum albumin (BSA-Sigma), 0.2% gelatine (Sigma) and 2% foetal bovine serum (Biowest) at RT. Staining for the HA-tag (mouse anti-HA) and/or lysosomal associated membrane protein 1 (rabbit anti-LAMP1) (Supplementary Table S2) was performed at 4 °C (overnight), followed by incubation with Alexa-labelled secondary antibodies and Hoechst 33342 for 45 min. Transfection efficiency was quantified as proportion of AF-488 positive cells after manual counting. HFM were also stained for the 3HA-tag, F-actin and LAMP1, according to previously described protocols^17^.

### Cystine measurement in vitro

Cystine levels were measured, as described previously^47^. Cells were lysed in 100 µl 50 mM *N*-ethylmaleimide (NEM-Sigma) and 50 µl of 12% sulfosalicylic acid (SSA-Sigma). Cells are lysed by centrifugation at high speed (13,000 rpm, 10 min) and the supernatant is isolated and stored at − 80 °C. Analysis of total cystine levels was performed in the Bambino Gesù Children’s Hospital. Values were normalized to total protein after a Bicinchoninic acid assay (Thermofisher) on the cell pellet.

### Quantification of lysosomal distribution

After immunostaining, the lysosomal signal was quantified in the perinuclear (< 25% of area around the nucleus) and peripheral region (> 25% of area around the nucleus). Lysosomal distribution was assessed in imageJ by calculating the % of LAMP1 intensity over the radius of the cell, by means of quantification of the area under the curve after application of the ‘plot profile’ tool illustrating the mean fluorescence for each pixel detected.

### FITC-inulin leakage assay

To quantify the tightness of the PTEC monolayer after culturing the cells on the hollow fiber membrane, the tubules were perfused with a FITC-inulin solution (0.1 mg/ml) as described previously^17^. After completing the perfusion, three technical replicate samples (100 µl) were taken from the apical chamber of each biological replicate and fluorescence measured with a Tecan infinite M200PRO plate reader (Tecan Austria GmbH, Grödig, Austria) using a 492 nm excitation wavelength and 518 nm emission wavelength.

### Fish maintenance

Zebrafish were handled and maintained in compliance with the KU Leuven animal welfare regulations (ethical approval 142/2019). Embryonic *ctns*^*−/−*^ (*ctns* c.706C>T, p.Q236X)^18^ and *ctns*^*−/−*^*[Tg(l-fabp:DBP:eGFP)]* were used below the age of 5 days post-fertilization (in accordance with the European Commission Directive 2010/63/EU and the commission implementing decision (EU) 2020/569). The *ctns*^*−/−*^*[Tg(l-fabp:DBP:eGFP)]* fish were established by crossing the *ctns*^+*/*+^*[Tg(l-fabp:DBP:eGFP)]* fish^19^, expressing a vitamin D-binding protein (DBP) fused with *eGFP* for detection of proteinuria, with the *ctns*^−/−^ zebrafish. Larvae were subsequently screened for green fluorescence at 5 days post-fertilization with the SteREO Discovery V8 microscope (Zeiss, Germany) and GFP positive larvae raised up. Genotyping was used to select *ctns*^*−/−*^*[Tg(l-fabp:DBP:eGFP)],* as described previously^18^, and the presence of *eGFP* was confirmed by PCR. All experiments were reported in accordance with the ARRIVE guidelines.

### Microinjection of mRNA preparations in zebrafish larvae

Human mCherry-tagged *CTNS* mRNA solutions were prepared in RNAse free water (500 ng/µl) with 0.05% PhenolRed (Sigma). All mRNA injections were performed at the one-cell stage using a FemtoJet microinjector (Thermofisher) and borosilicate glass microcapillaries (World Precision Instruments), prepared using a micropipette puller device (Sutter Instruments), or ready-made Eppendorf FemtoTips II. A tip opening diameter of ~ 2 µm was used and microinjections were set up with an injection volume of ~ 1.7 nl, resulting in a final concentration of ~ 0.85 ng mRNA per embryo.

### Toxicity studies in zebrafish

Embryonic health was evaluated at 5 days post-fertilization by quantification of percentage of embryonic death, and morphological abnormalities^18^. Unfertilized eggs were removed upon injection.

### Evaluation of *CTNS-mCherry* mRNA levels and protein expression in zebrafish

RNA levels after injection were determined by means of PCR at 24 h, 72 h and 120 h. Collected embryos were sonicated in 1 ml of Trizol (Thermofisher) and phase separation was performed with chloroform (Sigma), according to the manufacturer’s protocol. cDNA was synthesized with a mix of Oligo (dT) (Thermofisher), random primers (Thermofisher), dNTP mix (VWR) and SuperScript-III Reverse Transcriptase (Invitrogen), according to the manufacturer’s protocol. Quantitative PCR (annealing temperature = 60 °C, 40 cycles) was performed with 1× Platinum ® SYBR® Green (Thermofisher) and 0.2 µM *CTNS* or *bactin1* primers in a StepOne™ Real-Time PCR System (Supplementary Table S3)*.* Protein expression was quantified for randomly selected embryos based on fluorescence in a predetermined 200 × 100 pixels area in the head region and confirmed on western blot. Lysates were prepared from deyolked embryos by sonication with RIPA buffer (Thermofisher) and protease inhibitors (Roche). Protein content was determined with a Bicinchoninic acid assay (Thermofisher) and 50 µg was loaded onto a NuPAge 4–12% BisTris gel (Thermofisher). Proteins were transferred on a nitrocellulose membrane and incubated with anti-RFP (overnight, 4 °C). Bands were visualized with HRP-linked secondary antibodies and ECL Plus substrate (Thermofisher) in the Syngene Chemi XRQ System. Beta-actin was used as a loading control.

### Cystine measurement in zebrafish

Larvae were collected in groups of 10–12 fish at 72 and 120 h post-injection, washed with methylene blue-free aquarium water and homogenized in 200 µl of 50 mM NEM and 100 µl of 12% SSA. The homogenate is processed as described above^18^. Values were normalized to total protein after a Bicinchoninic acid assay (Thermofisher).

### Analysis of kidney function in *ctns*^*−/−*^*[Tg(l-fabp:DBP:eGFP)]* zebrafish

Tubular reabsorption was assessed after injection of 10 kDa Alexa-647 dextran (Sigma), as described^18^. 16 h after dextran injection, larvae were prepared for cryosectioning with the LeicaSM 200R cryomicrotome (6 µm, Leica, Germany), as described before^48^. Finally, slides were stained with Hoechst 33342 and 2 µg/ml CF770-conjugated wheat-germ agglutinin (biotium) for 10 min at RT.

Proteinuria of sham and *CTNS-mCherry* mRNA injected *ctns*^*−/−*^*[Tg(l-fabp:DBP:eGFP)]* larvae was assessed by DBP-GFP fluorescence. Larvae were mounted in 96-well plates and imaged on an Acquifer Imaging Machine (Acquifer Imaging GmbH, Germany). Intravascular fluorescence intensities of the tail region were quantified with an automated FIJI script, segmenting blood vessels upon traversing blood cells in a short image sequence and measuring GFP fluorescence within the masks^49^.

### Immunostaining of zebrafish larvae cryosections

Fish were fixed overnight in 4% PFA and washed with phosphate buffered saline (PBS-3x) and PBS with 0.1% Triton X-100 (Sigma). Larvae were kept overnight in 30% sucrose (Merck), followed by embedding in gelatine (Sigma) with 15% sucrose into a disposable mould (Electron microscopy sciences). Sections were made with the NX70 cryostat (4 µm, Thermofisher, USA) and blocked by overnight incubation in PBS + 0.1% Triton X-100 and 5% donkey (Abcam) or goat serum (DAKO), followed by incubation with primary antibody (overnight) and secondary Alexa-labelled antibody (2 h). Sections were stained with Hoechst 33342 and mounted with fluorescence mounting medium (DAKO).

### Cleaved caspase-3 staining on zebrafish paraffin sections

Four zebrafish embryos (120 h post-injection) were fixed in 4% PFA, transferred to 70% ethanol and embedded in paraffin, as described previously^18^. After antigen-retrieval, sections were incubated overnight at room temperature with anti-cleaved caspase-3 rabbit antibody (Supplementary Table S2). The cleaved caspase-3 was visualized with an anti-rabbit envision HRP-labelled antibody (Supplementary Table S2). The tubulus was identified based on the inclusion of a haematoxylin and eosin staining.

### Microscopy

Images were acquired on an Operetta CLS High Content Screening Microscope (Perkin-Elmer, Germany—VIB Bio Imaging Core Leuven, Belgium), Zeiss LSM 880-Airyscan (Cell and Tissue Imaging Cluster (CIC), KU Leuven, Belgium) and Eclipse CI microscope (Nikon, Japan). Zebrafish embryos were imaged with the Olympus IX71 widefield fluorescence microscope (Olympus—VIB bioimaging core Leuven, Belgium) and the SMZ18 fluorescence stereomicroscope with a P2-SHR Plan Apo 1× objective, motorized Z-drive (Nikon GMBH, Germany) and an X-Cite Xylis LED (Excelitas, Germany). 10 kDa dextran images were obtained with laser scanning confocal microscopy (LC-LSM). For studying of proteinuria, zebrafish were imaged with the Acquifer Imaging Machine.

### Statistical analysis

Data were analysed using GraphPad Prism version 8.0.0 for Windows (San Diego, California USA, www.graphpad.com). Normality was assessed by a QQ-plot and equality of variances with an F-test (Brown–Forsythe). When data was not normally distributed or n < 5, a non-parametric test was used. Average (± SEM) or median with 95% confidence interval (CI) [*x,x*] are presented for normal data and skewed data, respectively. Outliers were removed based on a ROUT outlier test (Q = 1%). Two groups were compared with a Student’s t-test or Mann–Whitney test, multiple groups with an one-way ANOVA or Kruskal Wallis test with Bonferroni or Dunn’s multiple testing, respectively. Equality of variances was tested by means of a Brown–Forsythe F-test, followed by Welch’s correction in the t-test or one-way ANOVA (followed by Dunnett’s multiple testing). A double-sided p-value < 0.05 was defined as being significant.

### Supplementary Information

Supplementary Information.

## Data Availability

The data that support the findings of this study can be found in the manuscript and Supplementary Data. The datasets generated and/or analysed during the current study are available from the corresponding author on reasonable request.
