# Supplementary material for: Evaluation of the efficacy of cystinosin supplementation through CTNS mRNA delivery in experimental models for cystinosis
Source: Sci Rep. 2023 Nov 28;13:20961. doi: 10.1038/s41598-023-47085-w (PMC10684520; doi:10.1038/s41598-023-47085-w)
Supplement: Supplementary file 1 — Supplementary Information. [file 41598_2023_47085_MOESM1_ESM.pdf]

Supplementary Materials: Evaluation of the efficacy of cystinosin supplementation through *CTNS* mRNA delivery in experimental models for cystinosis.

Tjessa Bondue[1], Sante Princiero Berlingerio[1+], Florian Siegerist[2+], Elena Sendino-Garví[3], Maximilian Schindler[2], Hans Jacobus Baelde[4], Sara Cairoli[5], Bianca Maria Goffredo[5], Fanny Oliveira Arcolino[1,6], Jürgen Dieker[7], Manoe Jacoba Janssen[3], Nicole Endlich[2], Roland Brock[8,9], Rik Gijsbers[10,11], Lambertus van den Heuvel[1,12++], Elena Levtchenko [1,13++ *]

*e.n.levtchenko@amsterdamumc.nl

[+] / [++] These authors contributed equally to this manuscript

1. Laboratory of Pediatric Nephrology, Department of Development and Regeneration, KU Leuven, Leuven, Belgium.
2. Institute of Anatomy and Cell Biology, Universitätsmedizin Greifswald, Greifswald, Germany
3. Division Pharmacology, Utrecht Institute for Pharmaceutical Sciences, Utrecht University, Utrecht, The Netherlands.
4. Department of Pathology, Leiden University Medical Center, Leiden, The Netherlands
5. Laboratory of Metabolic Biochemistry, Department of Pediatric Medicine, Bambino Gesù Children's Hospital, IRCCS, Rome, Italy
6. Department of Pediatric Nephrology, Emma Children's Hospital and Emma Center for Personalized Medicine, Amsterdam UMC, Amsterdam, The Netherlands
7. Mercurna B.V., Oss, The Netherlands
8. Department of Medical Biosciences, Radboud University Medical Center, Nijmegen, The Netherlands
9. Department of Medical Biochemistry, College of Medicine and Medical Sciences, Arabian Gulf University, Manama, Kingdom of Bahrain
10. Laboratory for Molecular Virology and Gene Therapy, Department of Pharmaceutical and Pharmacological Sciences, KU Leuven, Leuven, Belgium
11. Leuven Viral Vector Core (LVVC), KU Leuven, Leuven, Belgium
12. Department of Pediatric Nephrology, Radboud University Medical Center, Nijmegen, The Netherlands
13. Department of Pediatric Nephrology, Emma Children's Hospital, Amsterdam UMC, Amsterdam, The Netherlands.

Supplementary Table S1: Transfection efficiencies in cells used for intracellular cystine measurement.

	**Proportion of cells with detectable cystinosin-3HA protein expression***	
**mRNA dose**	**Proximal tubular epithelial cells**	**Podocytes**
**83ng/ml *CTNS-3HA* mRNA**	18%	57%
**250ng/ml *CTNS-3HA* mRNA**	63%	67%
**500ng/ml *CTNS-3HA* mRNA**	87%	86%

***** Representative numbers are shown, derived from a single experiment.

Supplementary Table S2: List of antibodies used in this study.

**Primary antibody**	**Manufacturer**	**Dilution**
Anti-HA (mouse)	BioLegend, cat.no. 901515	1:1000
Anti-LAMP1 (rabbit)	Cell signalling, cat.no. 9091S	1:1000
Anti-(zebrafish)megalin (rabbit)	Homemade (Manchester, Prof. Martin Lowe)	1:200
Anti-RFP (rabbit)	Rockland, cat.no. 600-401-379	1:200 (IF), 1:1000 (WB)
Anti-Actin (mouse)	Sigma, cat.no. A5441	1:5000
Anti-cleaved caspase-3	Cell signalling, cat.no. 9661S	1:100
**Secondary antibody**	**Manufacturer**	**Dilution**
Hoechst 33342	Thermofisher Scientific, cat.no. 62249	1:1000
Goat anti-Rabbit AF546	Thermofisher Scientific, cat.no. A11035	1:1000
Goat anti-Mouse AF488	Abcam, cat.no. ab150113	1:500
Donkey anti-Rabbit AF488	Abcam, cat.no. ab150073	1:500
Phalloidin AF647	Thermofisher Scientific, cat.no A30107	1:1000
Goat anti-Rabbit Immunoglobulins/HRP	Dako, cat.no. P044801-2	1:2000
Goat anti-Mouse Immunoglobulins/HRP	Dako, cat.no. P0447	1:2000
Envision anti-Rabbit HRP	Dako, cat.no. K4003	ND

The anti-RFP (=red fluorescent protein) antibody was used for CTNS-mCherry detection. LAMP1 = lysosomal associated membrane protein 1. AF = Alexa Fluor, HRP = horseradish peroxidase, ND = not diluted

Supplementary Table S3: List of primers used in this study**.**

Gene	Forward Primer (5' -> 3')	Reverse Primer (5' -> 3')	Size of the expected band (bp)
CTNS (human - NM_004937.3)	CAGCGCCATTAGCATCATAAA (exon 7)	GAAACTGCTCCTTGATGTA (exon 8-9 spanning)	212
bactin 1 (zebrafish - AF057040.1)	ACGGTCAGGTCATCACCAT	AGGGTACATGGTGGTACCTC	191

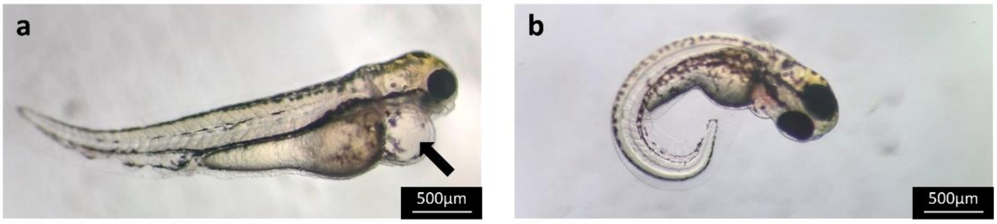

Supplementary Figure S1: Assessment of mRNA toxicity in *ctns*$^{-/-}$ zebrafish.

For the evaluation of toxicity following *CTNS-mCherry* mRNA injection, morphology of larvae was assessed at 120h post-injection. Two types of dysmorphism were considered, larvae presenting with pericardial oedema (a - arrow) and/or a curved spine (b). Scale bar = 500µm

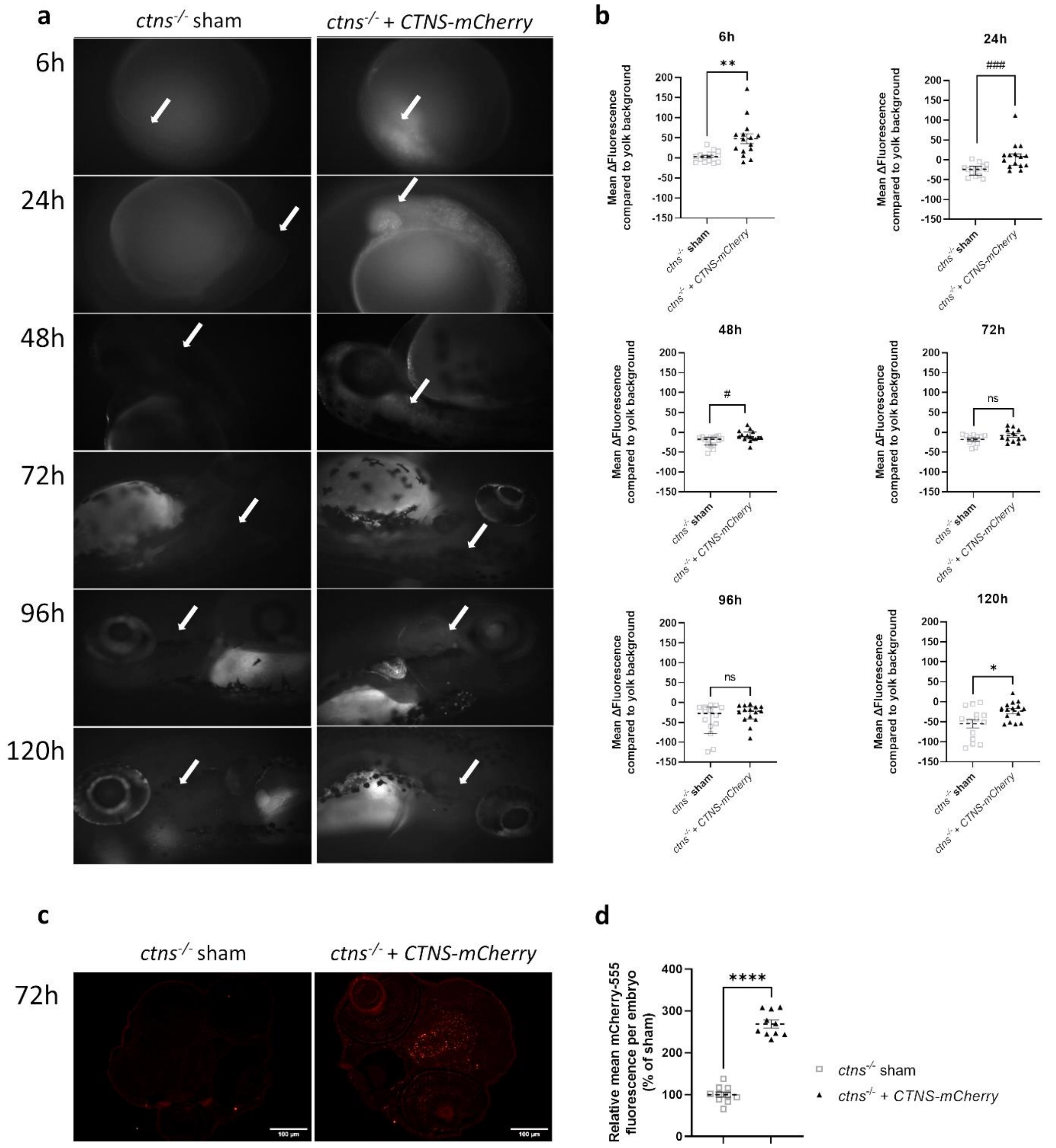

Supplementary Figure S2: Injection of *CTNS-mCherry* mRNA in fertilized eggs of *ctns*$^{-/-}$ zebrafish results in embryonic protein expression for up to 72h.

(a) Cystinosin-mCherry expression was assessed at 6h, 24h, 48h, 72h, 96h and 120h post-injection in the live embryos, and mRNA treated fish were compared with the sham treated control. Images were taken using the Olympus IX71 widefield microscope. (b) Quantification of mean cystinosin-mCherry fluorescence in the head region of each fish after yolk signal subtraction. Significance was tested for each time point by means of Mann-Whitney test (#, Median and 95% CI are shown)) or t-test (*, mean and SEM are shown). */#, $P < 0.05$; **, $P < 0.01$; ###, $P < 0.001$. (c) In zebrafish cryosections, the mCherry-tag could also be detected by means of immunostaining using anti-mCherry antibodies in 72h old zebrafish. Images were obtained with the Nikon Eclipse CI microscope. Scale bar = 100µm. (d) Mean fluorescence per head was quantified and significance was tested compared with sham injected fish (n = 4 fish, with multiple sections analysed per fish) by means of Student's t-test. Mean and SEM are shown. ****, $P < 0.0001$.

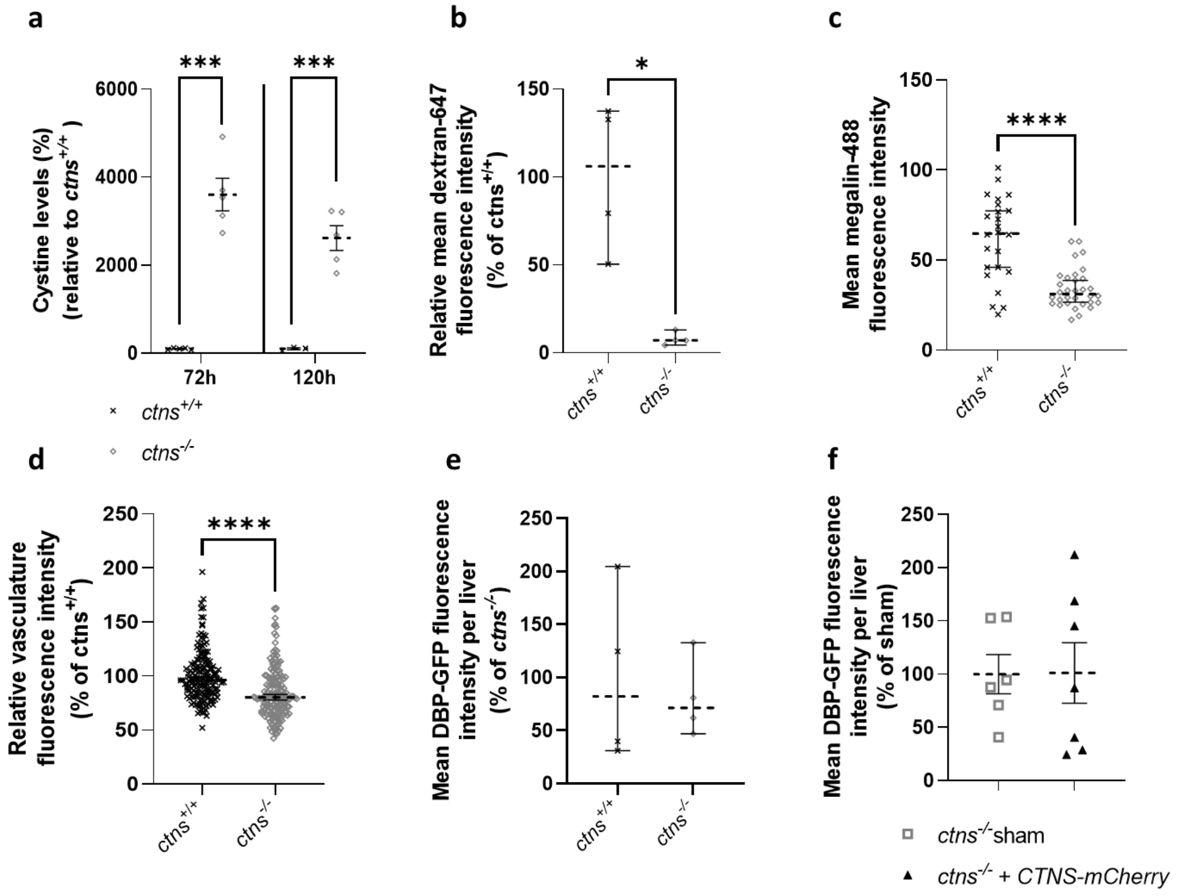

Supplementary Figure S3: *ctns*$^{-/-}$ fish accumulate cystine and *ctns*$^{-/-}$*[Tg(l-fabp:DBP:eGFP)]* zebrafish present with proximal tubular dysfunction and proteinuria.

(a) Cystine levels in *ctns*$^{+/+}$and *ctns*$^{-/-}$ larvae were measured at 72h and 120h post-fertilization. Statistical analysis was performed using a Welch's t-test. Mean and SEM are indicated. ***, P < 0.001. (b) *ctns*$^{-/-}$*[Tg(l-fabp:DBP:eGFP)]* and *ctns*$^{+/+}$*[Tg(l-fabp:DBP:eGFP)]* larvae were injected with 10kDa dextran-AF647 for evaluation of low molecular weight proteinuria (LMWP) at 72h post-injection and fixed for cryosection after 16h. Tubular intensity of dextran-AF647 fluorescence (magenta) was measured and used to quantify low molecular weight protein absorption. Data were analysed with a Mann-Whitney test and median with 95% CI is indicated (n=4 fish). Scale bar = 100µm. (c) Alexa-488 megalin intensity was measured in tubules of in 72h post-fertilization *ctns*$^{+/+}$and *ctns*$^{-/-}$ larvae. Statistical significance was tested using an Mann-Whitney test and median with 95% CI is indicated. ****, P < 0.0001. (d) Proteinuria was demonstrated in *ctns*$^{-/-}$*[Tg(l-fabp:DBP:eGFP)]* larvae by measurement of reduced relative vasculature fluorescence intensity in comparison with the wildtype *ctns*$^{+/+}$ *[Tg(l-fabp:DBP:eGFP)]* larvae. Images were generated with the Acquifer imaging machine. Data were analysed with a Mann-Whitney test (n = 166 and 176 respectively), with median and 95%CI indicated. ****, P < 0.0001. (e) DBP-eGFP fluorescence was quantified in *ctns*$^{+/+}$ and *ctns*$^{-/-}$ fish to confirm comparable baseline expression of the protein. Statistical significance was tested with a Mann-Whitney test and median with 95% CI is indicated. (f) Mean DBP-eGFP fluorescence was also quantified in the livers of sham injected and *CTNS-mCherry* injected fish. Statistical significance was tested with a Student's t-test and mean with SEM is indicated.

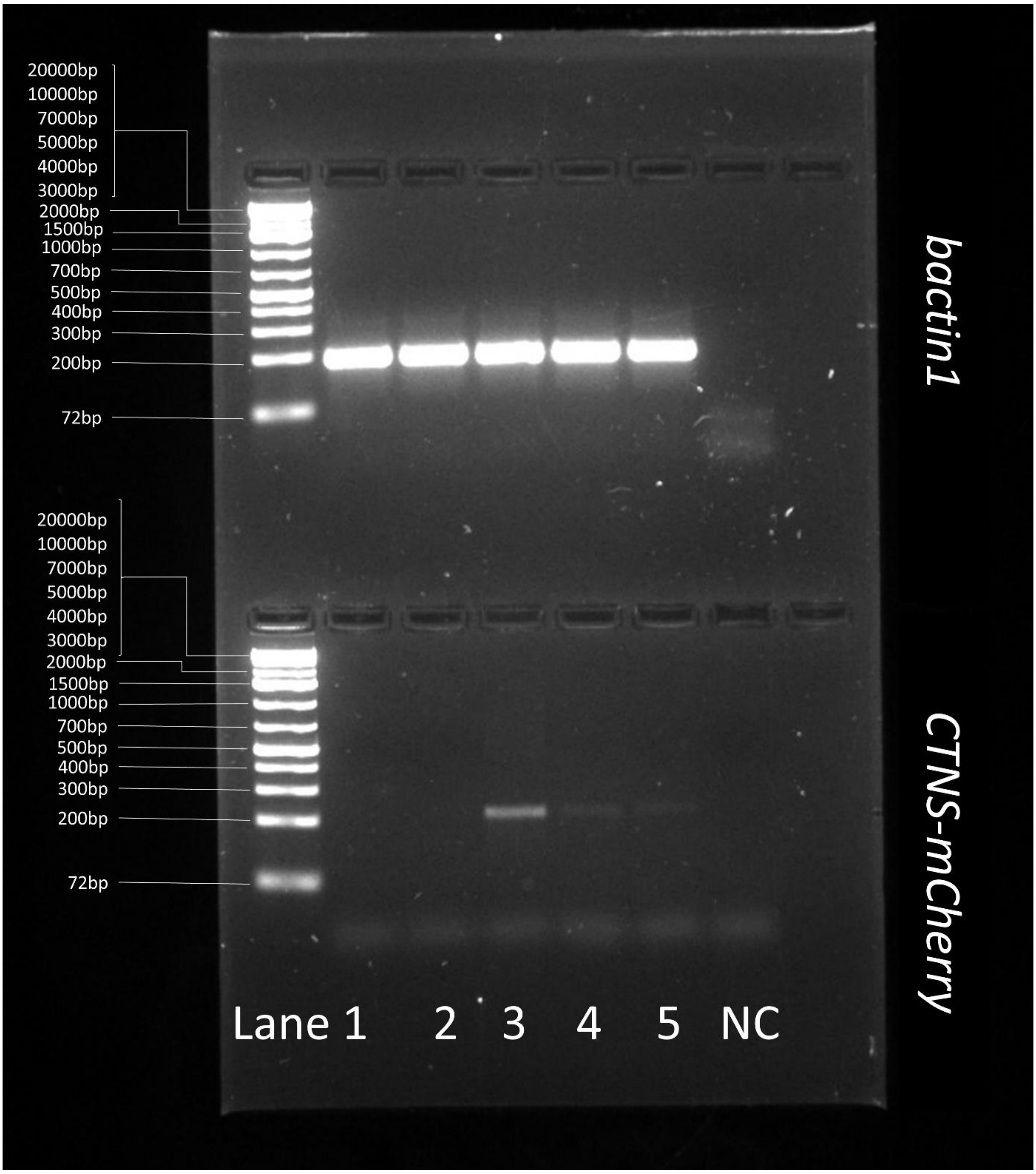

Supplementary Figure S4: Uncropped and labelled gel as displayed in Figure 3c.

CTNS-mCherry mRNA levels were shown in the untreated (lane 1) and sham injected (lane 2) fish and at 24h (lane 3), 72h (lane 4) and 120h (lane 5) post-injection by agarose gel electrophoresis. Expression of bactin1 mRNA (top) was used for normalization. The GeneRuler 1 kb Plus DNA Ladder (Thermofisher , cat.no.SM1333) was used as a size marker. bp=basepairs

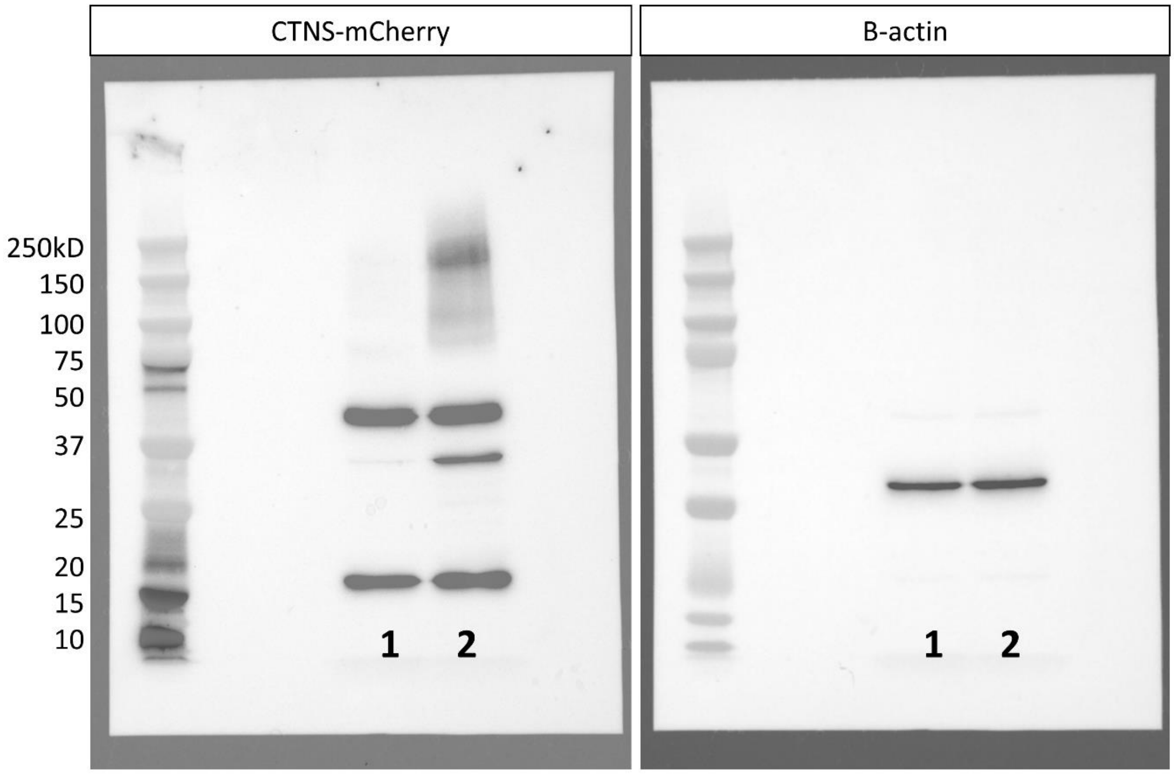

Supplementary Figure S5: Uncropped and labelled western blot as displayed in Figure 3g.

Expression of cystinosin-mCherry protein at 24h was confirmed in *CTNS-mCherry* injected fish (Lane 2, >70kD, glycosylated) in comparison with the sham injected control (Lane 1 (=negative control)) by a western blot (top) with beta-actin as the loading control (bottom). As a molecular weight marker, the Precision plus protein dual color standards (Biorad, cat.no.161-0374) was used. kD = kilodaltons
